# Supplementary material for: Campsites, forest fires, and entry point distance affect earthworm abundance in the Boundary Waters Canoe Area Wilderness
Source: PeerJ. 2020 Feb 25;8:e8656. doi: 10.7717/peerj.8656 (PMC7047863; doi:10.7717/peerj.8656)
Supplement: Supplemental Information 2 — Figure 1: Residuals from the SEM compared across the lakes. Figure 2: Variogram of residuals from the SEM. The plot shows no evidence for spatially auto-correlated residuals, indicating samples from same lakes can be treated as independent. [file peerj-08-8656-s002.pdf]

Figure I (supplemental)

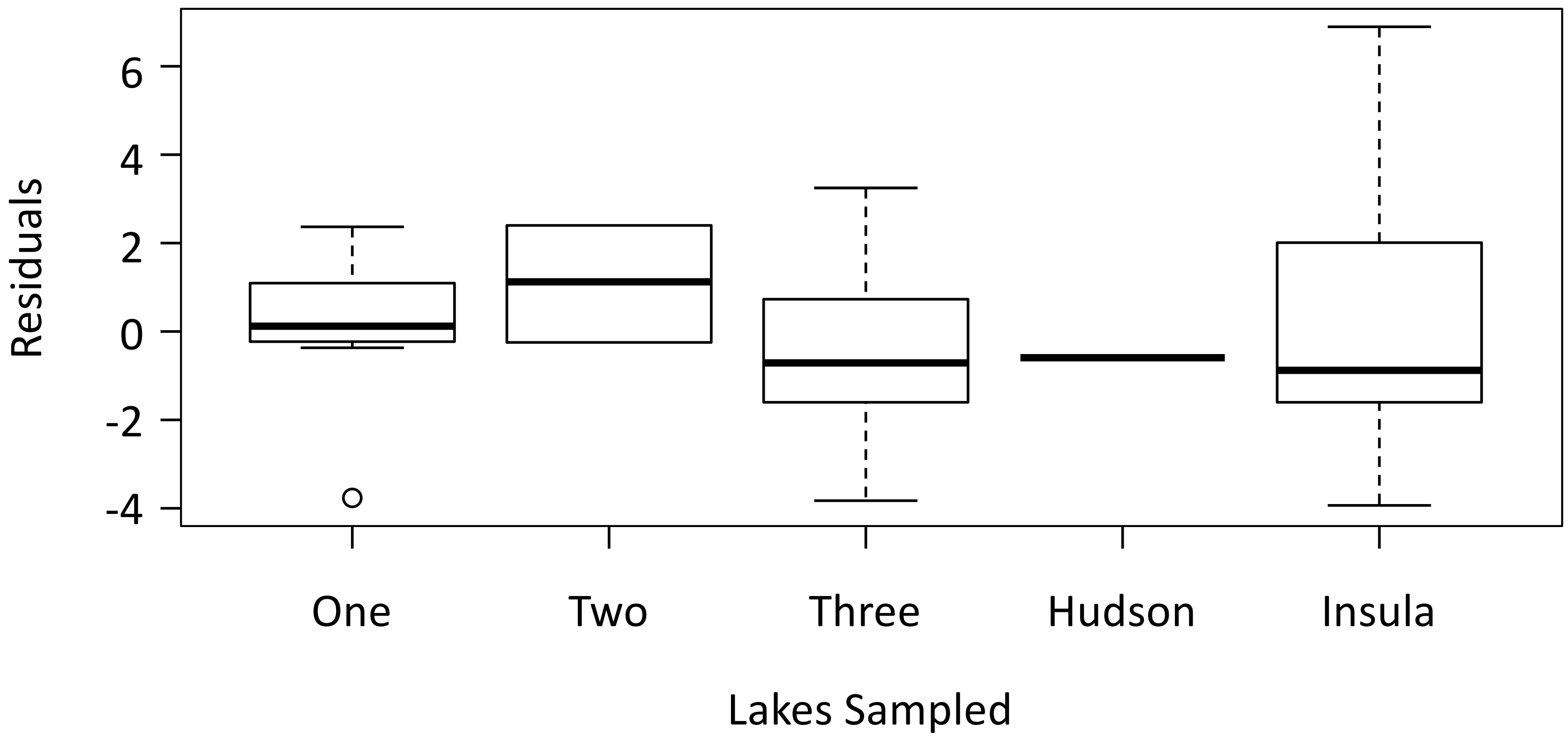

Residuals from the SEM compared across the lakes.

Figure II (supplemental)

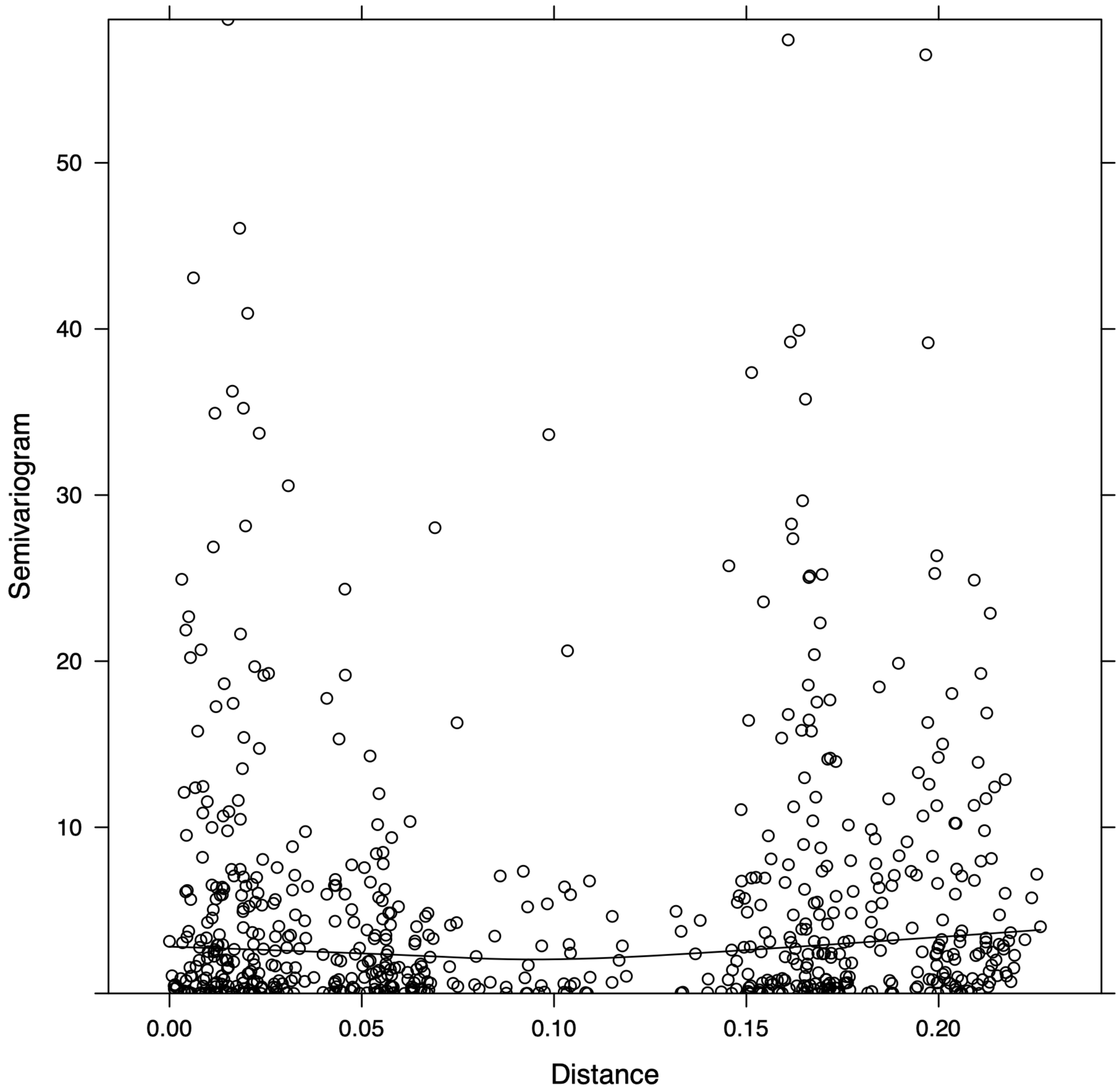

Variogram of residuals from the SEM. The plot shows no evidence for spatially auto-correlated residuals, indicating samples from same lakes can be treated as independent.
